# Adsorption and Desorption Characteristics of Total Flavonoids from *Acanthopanax senticosus* on Macroporous Adsorption Resins

**DOI:** 10.3390/molecules26144162

**Published:** 2021-07-08

**Authors:** Xiaoya Wang, Jianqing Su, Xiuling Chu, Xinyu Zhang, Qibin Kan, Ruixue Liu, Xiang Fu

**Affiliations:** College of Agronomy, Liaocheng University, Liaocheng 252000, China; wangxy9625@163.com (X.W.); xinxinxinxinyuyuyu@163.com (X.Z.); kanqibin@163.com (Q.K.); liuruixue919@163.com (R.L.); fuxiang2499570264@163.com (X.F.)

**Keywords:** macroporous resin, *Acanthopanax senticosus*, total flavonoids, adsorption characteristics

## Abstract

We examined the application of six different resins with the aim of selecting a macroporous resin suitable for purifying *Acanthopanax senticosus* total flavonoids (ASTFs) from *Acanthopanax senticosus* crude extract (EAS) by comparing their adsorption/desorption capacities, which led to the selection of HPD-600. Research on the adsorption mechanism showed that the adsorption process had pseudo-second-order kinetics and fit the Freundlich adsorption model. Moreover, the analysis of thermodynamic parameters indicated that the adsorption process is spontaneous and endothermic. The optimal conditions for purification of ASTFs were determined as sample pH of 3, 60% ethanol concentration, and 3 BV·h^−1^ flow rate, for both adsorption and desorption, using volumes of 2.5 and 4 BV, respectively. The application of macroporous resin HPD-600 to enrich ASTFs resulted in an increase in the purity of total flavonoids, from 28.79% to 50.57%. Additionally, the antioxidant capacity of ASTFs was higher than that of EAS, but both were lower than that of L-ascorbic acid. The changes in ASTFs compositions were determined using ultra-performance liquid chromatography–tandem mass spectrometry (UHPLC–MS/MS), with the results illustrating that the levels of seven major flavonoids of ASTFs were increased compared to that in the crude extract.

## 1. Introduction

*Acanthopanax senticosus* (AS) belongs to the family Araliacea, which is mainly distributed throughout China, Japan, and South Korea [1]. The main regions where it is produced are Liaoning, Jilin, Heilongjiang, Hebei, and Shanxi in China. The root, stem, leaf, flower, and fruit of AS all have medicinal value, and the Chinese Pharmacopoeia stipulates that its dry root, rhizome, and stem are medicines. AS is not only used as herbal medicines but also as function foods, such as AS tea, AS wine, etc. [2]. AS has the effect of tonifying qi and the entire body, particularly tendons and bones. It is mainly used to treat spleen and kidney yang deficiency, body deficiency fatigue, loss of appetite, waist and knee pain, insomnia, and bad dreams [3]. The active components of AS include glycosides, polysaccharides, flavonoids, lignans, triterpenoids, and organic acids [4]. Flavonoids are one of the main effective components of AS, which have two forms: one is a free state, and the other is glycosides combined with carbohydrates. Most of the flavonoids in AS are found in the latter state. Many research articles have demonstrated the sedative, antioxidant, anti-inflammatory, and anti-aging effects of ASTFs [5,6]. Its composition is complex, containing some sugars and other impurities that cannot be related to its beneficial effects or may even have undesirable effects. It is necessary to separate and purify ASTFs as the active components for medicinal application. At present, the existing methods for separating flavonoids include supercritical fluid extraction, organic solvent extraction, and so on, but the disadvantages of these methods are high solvent consumption, time-consuming, low separation efficient, or the solvents are harmful to environment or humans. Macroporous resin is prepared by polymerization reaction of various additives, such as polymerized monomer and crosslinking agent, porogen, dispersant, etc. It has the advantages of low costs [7], great adsorption performance [8], high reuse rate, good safety [9], and fewer interfering factors [10]. Macroporous resins are generally divided into three categories: non-polar, weak polar, and polar macroporous resins. Macroporous resins can selectively adsorb the targeted constituents from aqueous and nonaqueous system through Van der Waals force, hydrogen bonding interaction, electrostatic force, complexation, and size sieving action [11,12]. Adsorption and desorption are not only affected by polarity but also by the surface area and pore size of the macroporous resin. The use of macroporous resin adsorption as an effective method to purify total flavonoids has been reported in the separation and purification of flavonoids, such as *Sophora tonkinensis Gagnep* [13], *Ginkgo biloba leaves* [14], *Rhizoma smilacis glabrae* [15], and *Pteris ensiformis Burm* [16]. This experiment enriches the total flavonoids of *Acanthopanax senticosus* and understands its adsorption mechanism through the selection of macroporous resin and single factor investigation, studying adsorption kinetics, adsorption isotherms and thermodynamics, etc. In addition, four methods of measuring the antioxidant capacity of DPPH, ABTS, hydrogen radical, and reducing power were used to compare the antioxidant capacity of the total flavonoids of *Acanthopanax senticosus* before and after purification. It is hoped that this result can provide a theoretical and scientific basis for improving the utility of ASTFs.

## 2. Results and Discussion

### 2.1. Determination of Total Flavonoids Content

The total flavonoid content was calculated using a standard curve (*Y* = 0.0109*X* − 0.0077); and the linear correlation coefficient was *R*^2^ = 0.9992. The content of total flavonoids was measured based on rutin as the substance. Because total flavonoids are compounds, its content cannot be directly determined. The basic structure of flavonoids is a conjugated systems of A ring and B ring, and the structure of rutin monomer is fully satisfied, and it is obvious that the UV absorption of the conjugated systems. Hence, rutin is used as a representative to determine the content of flavonoids in many articles [17].

### 2.2. Selection of Macroporous Resins

**Figure 1:**
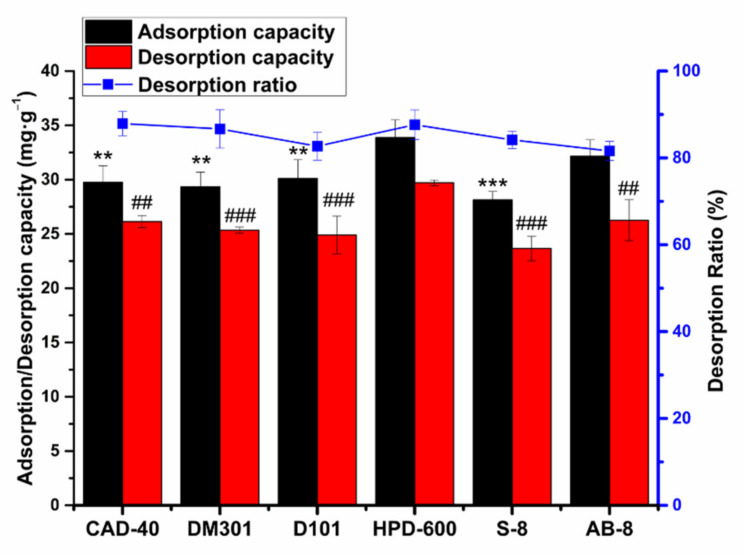
Adsorption, desorption capacity (mg·g^−1^), and desorption ratio (%) of ASTFs on the six different resins. Values are the mean ± SD of three independent repeated experiments. All saliency is compared with HPD-600. ** *p* ≤ 0.01 and *** *p* ≤ 0.001; ## *p* ≤ 0.01 and ### *p* ≤ 0.001.

The optimum macroporous resin was determined by investigating the adsorption/desorption ratio and capacity of six macroporous resins. The fundamental properties of the macroporous resins are listed in Table 1, with experimental results shown in Figure 1. In the present experiment condition, the data show that HPD-600 macroporous resin has the highest adsorption capacity of total flavonoids of *Acanthopanax senticosus* among the six tested resins. It is possible that one of the other resins could have outperformed HPD-600 under different conditions. In terms of desorption, the desorption capacity and desorption ratio of the HPD-600 resin were significantly higher than other resins (*p* < 0.01). Accordingly, the HPD-600 macroporous resin was selected for further purifying ASTFs due to its superior adsorption and desorption among the six resins. HPD-600 was more suitable to enrich flavonoids based on the fact that HPD-600 resin is a polar resin, and the flavonoids was polar compounds; it involves a principle “like dissolve like” [16]. And there are more hydrogen bonds and stronger van der Waals interactions between HPD-600 resin and *Acanthopanax senticosus* total flavonoids. In addition, HPD-600 has the biggest surface area in the six kinds of resins, indicating that the larger surface area can promote adsorption and desorption.

### 2.3. Adsorption Kinetics on HPD-600 Resin

The study of adsorption kinetics can detect the adsorption situation at each time point, view the adsorption process dynamically, and observe the change of adsorption rate and the adsorption equilibrium time during the adsorption process. Fitting the adsorption kinetic model can describe the relationship between adsorption rate and time, understand the diffusion of ASTFs on HPD-600 resin, and predict the adsorption results.

**Figure 2:**
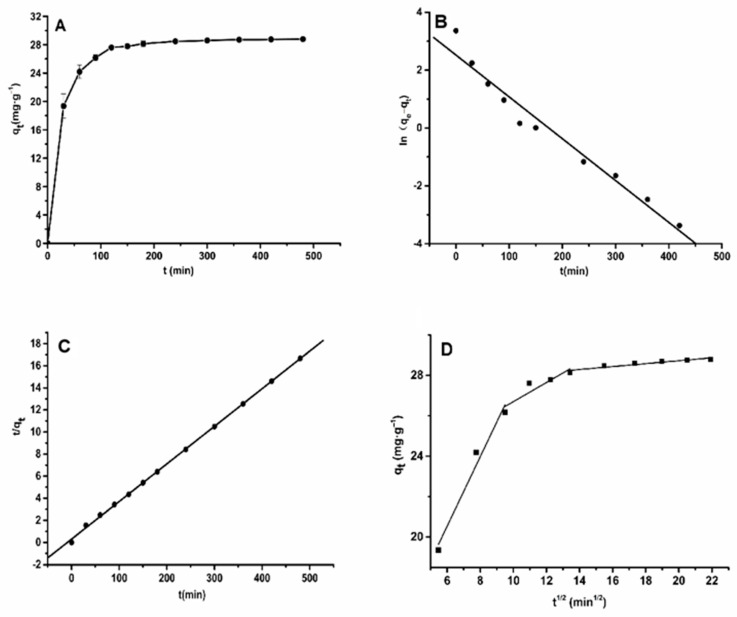
Adsorption kinetic curve (**A**) and linear correlations based on the pseudo-first-order (**B**), pseudo-second-order (**C**), and intra-particle diffusion (**D**) models for purifying ASTFs on the HPD-600 resin at 30 °C.

A kinetic curve was plotted according to the experimental data. In Figure 2A, it can be seen that the adsorption quantity increased with the increase in time in the aqueous solution, while the adsorption ratio was faster in the initial stage of adsorption. When the time reached 120 min, and the adsorption had likely achieved a saturated status, while the adsorption ratio tended to become slower. After that, even as the time increased, the adsorption of ASTFs on HPD-600 macroporous resin no longer changed, as the surface binding sites of macroporous resin had mostly been saturated with total flavonoids. Therefore, the equilibrium time for the adsorption of ASTFs on HPD-600 macroporous resin was 120 min in an aqueous solution. According to the method of 1.2.5, the data for the adsorption kinetic curve of ASTFs were separately fit to pseudo-first-order, pseudo-second-order kinetic models and particle diffusion, and the results are presented in Figure 2B–D. The fitting parameters in Figure 2 and Table 2 show that the adsorption kinetic data did not suitably fit to the particle diffusion and quasi-first-order kinetic models, with *R*^2^ values of 0.8514–0.9739 and 0.9616, respectively. The data were utilized to fit a quasi-second-order kinetic model with very good linear correlation indicated by an *R*^2^ of 0.9995. Moreover, the *k*_2_, *q_e_*, and *R*^2^ obtained from the quasi-first-order curve of adsorption are shown in Table 2. The theoretical adsorption quantity was 29.326 mg·g^−1^, which is close to the value of the experimental data (28.7863 mg·g^−1^). Therefore, the quasi-second-order kinetic model was more favorable for describing the adsorption mechanism in the process of purifying ASTFs, which indicates that HPD-600 adsorption of ASTFs occurs as a multilayer adsorption process.

### 2.4. Adsorption Isotherms on HPD-600 Resin

**Figure 3:**
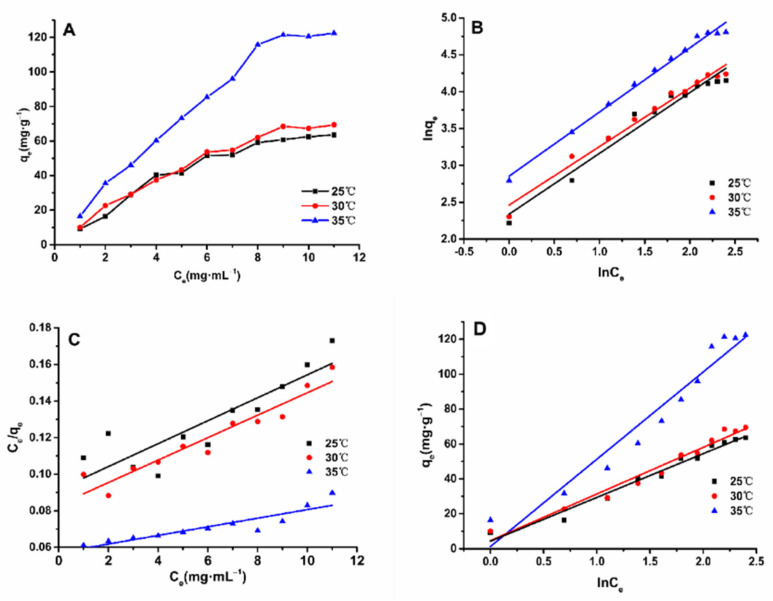
Adsorption isotherms (**A**) fitted by the Langmuir (**B**), Freundlich (**C**), and Temkin (**D**) models for purifying ASTFs using HPD-600 resin at 25, 30, and 35 °C.

Regarding adsorption isotherms, the relationship between the solute in solution and the macroporous resin when the solute is adsorbed on the macroporous resin and when the two substances reach a balance is known for a given temperature [18]. Based on the data obtained from the experiment, the adsorption isotherms of ASTFs on HPD-600 macroporous resin are shown in Figure 3. As shown in Figure 3, at the same temperature, the adsorption quantity increased rapidly when the total flavonoids were at a low concentration, but the adsorption ratio tended to flatten when the initial concentration of ASTFs was increased to 9 mg·mL^−1^. This phenomenon may be due to the fact that the resin surface became saturated with ASTFs due to the constant increase in ASTFs concentration [19]. The higher the experimental temperature, the stronger the adsorption capacity, which indicates that high temperatures are beneficial to the adsorption process, which is an endothermic reaction. 

Linear regression lines for adsorption isotherm models were plotted, including for Freundlich (Figure 3B), Langmuir (Figure 3C), and Temkin (Figure 3D) models. In Table 3, the isothermal model parameters are shown. The regression coefficient (*R*^2^ = 0.9772–0.9908) of the Freundlich model was the highest within the experimental temperature range, which fits the experimental data best. All these results indicate that the adsorption of ASTFs on HPD-600 macroporous resin shows heterogeneous behavior in monolayer and multilayer films [20].

The *K_F_* of the Freundlich model is related to adhesion ability. When the adsorption temperature increased from 25 to 35 °C, the *K_F_* value increased from 9.926 (mg·g^−1^) (mL·mg^−1^)^1/*n*^ to 17.354 (mg·g^−1^) (mL·mg^−1^)^1/*n*^, indicating that the increase in temperature enhanced the adhesion power of ASTFs on HPD-600 resin [21]. The exponential *n* is a heterogeneous factor and an indicator of the degree of nonlinearity of adsorption isotherms. It is generally believed that the larger value for parameter *n*, the better the adsorption effect. At 1 < *n* < 10, the adsorption is preferential; at *n* < 1, the adsorption effect is extremely poor, and continued reaction was unfavorable [22]. For the Freundlich model, the values of *n* exceeded 1, indicating that the adsorption process of flavonoids on HPD-600 is easy to continue, and that the adsorption process involved a physical process [23].

### 2.5. Adsorption Thermodynamics on HPD-600 Resin

**Figure 4:**
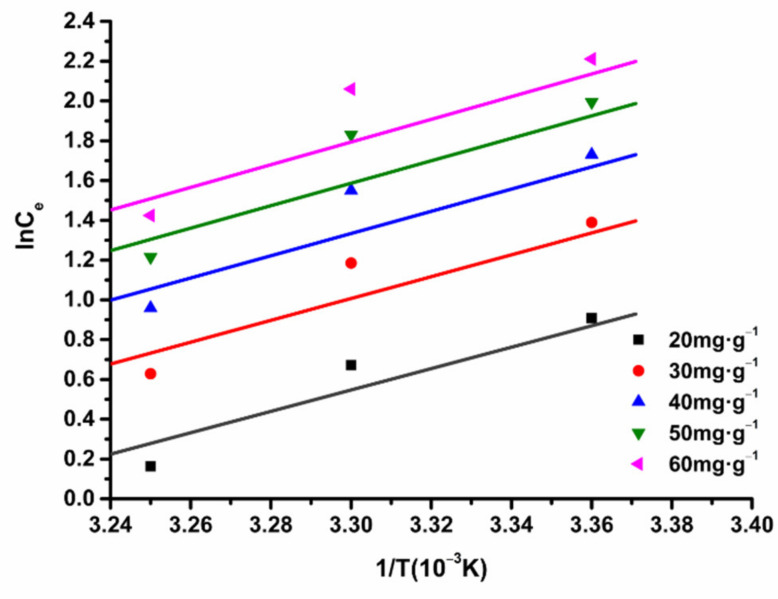
HPD-600 thermodynamic curve of total flavones with resin adsorption.

We used Equation (11) to fit ln*C_e_* to 1/T, and the results are shown in Figure 4. According to the slope of each fitting line in Figure 4, we calculated the enthalpy change ΔH. The characteristic parameters of the Freundlich adsorption isotherm model are shown in Table 4. Using Equation (12), we calculated the free energy ΔG. Finally, the entropy ΔS was calculated according to Equation (13), with results shown in Table 4. It can be seen that ΔH exceeds 0 at an equal adsorption quantity, which indicates that the process of enriching ASTFs on HPD-600 macroporous resin is endothermic, ΔH > 43 KJ·mol^−1^, suggesting that the adsorption process of ASTFs on HPD-600 perhaps involves a chemical process [24].

Meanwhile, the five straight lines are almost parallel, and the ΔH does not change obviously, at different adsorption capacities, which indicates that the effect of temperature on ΔH is not obvious. The result is in accordance with the conclusion that ΔH is assumed to be independent of T in the derivation of the Clausius–Clapeyron equation [25].

ΔG < 0, suggesting that the adsorption of ASTFs by HPD-600 resin was spontaneous. ΔS > 0, suggesting that the ASTFs were not restricted in adsorption to the HPD-600 resin. There solvent desorption was still exerted, and the entropy of this system increased.

### 2.6. Static Adsorption and Desorption Experiments

#### 2.6.1. The Influence of pH on Adsorption Capacity and Ratio

**Figure 5:**
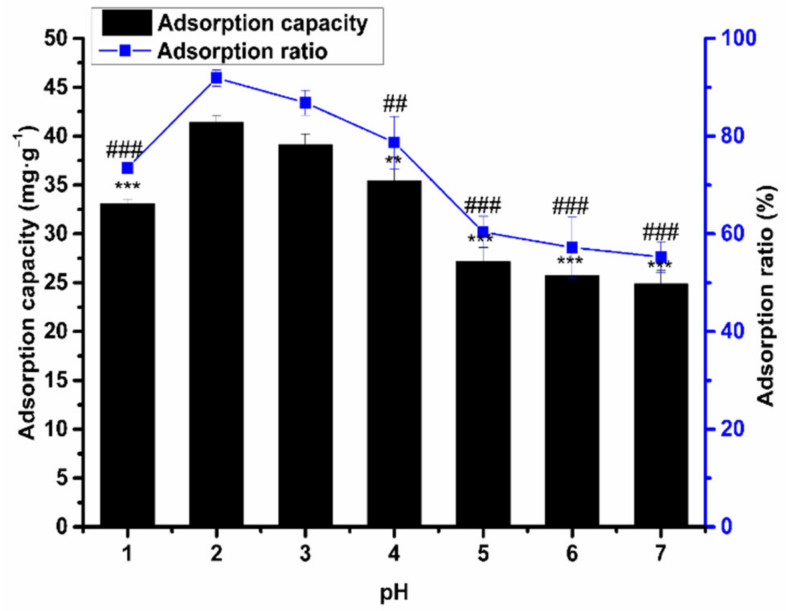
The influence of pH on the adsorption capacity/ratio of the HPD-600 resin. Values are the mean ± SD of three independent repeated experiments. All saliency is compared with the adsorption capacity/ratio of pH 2. ** *p* ≤ 0.01 and *** *p* ≤ 0.001; ## *p* ≤ 0.01 and ### *p* ≤ 0.001.

The adsorption quantity of the macroporous resin was largely affected by the sample solution pH, which is related to the surface charge characteristics and the ionization degree of the adsorbent [26]. Since flavonoids have a certain acidity and alkalinity, their solubility in solutions with different pH values is different, so the effect of pH on the adsorption capacity was studied. As shown in Figure 5, it can be seen that, when the pH increases from 1 to 2, the adsorption ratio raises, too. However, with the increase in pH above 2, the adsorption ratio of HPD-600 macroporous resin began to decrease. At pH 2, the adsorption ratio is at its maximum, but there is no significant difference compared with the adsorption ratio at pH 3. This result explains that the adsorption of AS total flavonoids on HPD-600 resins was suitable in higher pH. This may be because of the hydrogen-bonding interactions increasing at acidic conditions.

Moreover, if the pH is too low or too high, the molecular dissociation ability of the flavonoids will be weakened, and the adsorption ratio will be reduced. Therefore, pH 3 was selected for the optimal purification of the sample solution.

#### 2.6.2. The Influence of Ethanol Concentration on Desorption Capacity and Ratio

**Figure 6:**
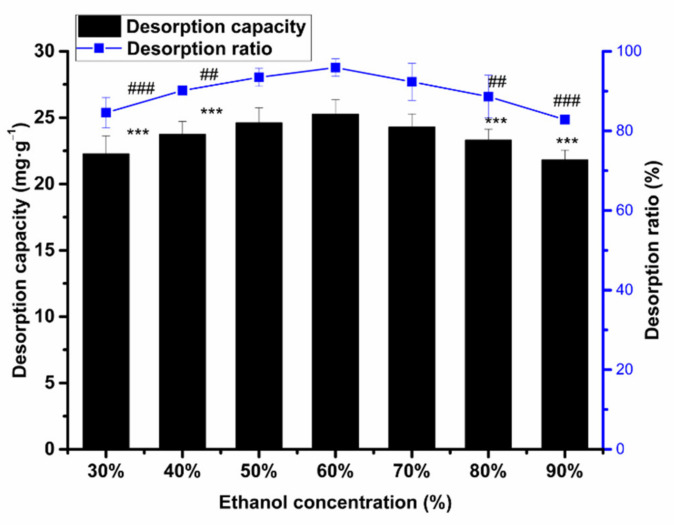
The influence of ethanol concentration on the desorption capacity/ratio of HPD-600 resin. Values are the mean ± SD of three independent repeated experiments. All saliency is compared with the desorption capacity/ratio of 60% ethanol. *** *p* ≤ 0.001; ## *p* ≤ 0.01 and ### *p* ≤ 0.001.

The total flavonoids can be desorbed by ethanol, for it can cripple the intermolecular forces between resin and total flavonoids, thus having total flavonoids enter the ethanol solution. We can see that the ratio of desorption first increases with the increase in ethanol concentration. The desorption ratio reached its maximum value, which was 95.91% at an ethanol concentration of 60%, as seen in Figure 6. Then, the desorption ratio showed a downward trend. Considering that the 60% ethanol solution had a low polarity and strong hydrogen bonding interaction with flavonoids, the concentration of the desorption solvent was 60% in the subsequent test.

### 2.7. Dynamic Adsorption and Desorption Experiments

#### 2.7.1. Effect of Adsorption Flow Rate on Adsorption Capacity

The plotting of the leakage curve is to investigate the leakage point; it is generally believed that, when the effluent concentration is 10% of the sample concentration, it means that the leakage point appears, and the adsorption process can be terminated in this time, and the sample volume is determined. Furthermore, the effect of different adsorption flow rate on the dynamic adsorption process was investigated because the ability of macroporous resin to adsorb the ASTFs at different flow rates and the required time are different.

**Figure 7:**
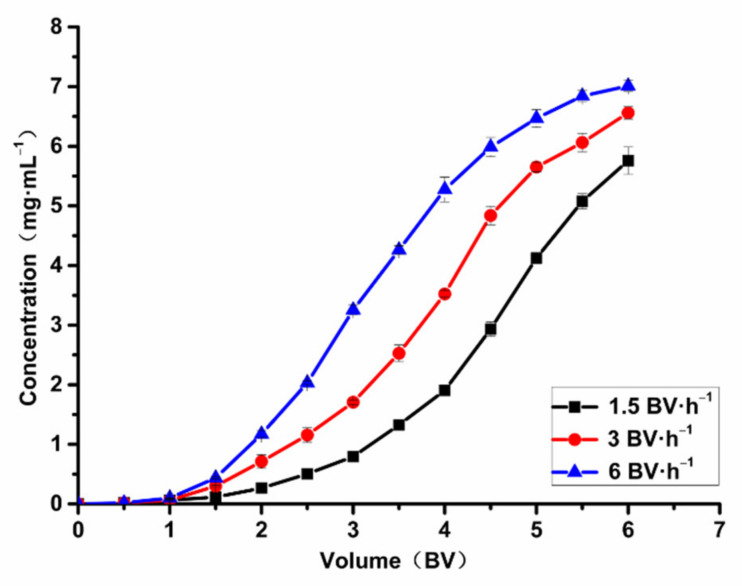
The influence of adsorption flow rate on adsorption capacity.

In Figure 7, the flow rate and sample volume are shown together. Figure 7 shows that the concentration of total flavonoids in the effluent increased gradually at three different flow rates, and then the increasing trend slowed down. The adsorption efficiency of ASTFs on HPD-600 resin decreased when the sample flow rate became larger. The total flavonoids were heavily absorbed at sample volumes of 3.5, 2.5, and 2 BV, at flow rates of 1.5, 3, and 6 BV·h^−1^. It can be seen from Figure 7 that the leakage point was reached earlier, and the competitive adsorption ratio decreased, when the flow rate was 6 BV·h^−1^, likely due to the shorter contact time and saturation [27]. When flow rate was 1.5 BV·h^−1^ and 3 BV·h^−1^, the leakage rate appeared late, and the concentration of effluent was always lower than 6 BV·h^−1^, which indicated that the adsorption effect was better at 1.5 BV·h^−1^ and 3 BV·h^−1^.Considering the time saved and adsorption effect, the optimal flow rate for purification of the sample was 3 BV·h^−1^, and the sample volume was 2.5 BV.

#### 2.7.2. The Influence of Desorption Flow Rate on Desorption Capacity

**Figure 8:**
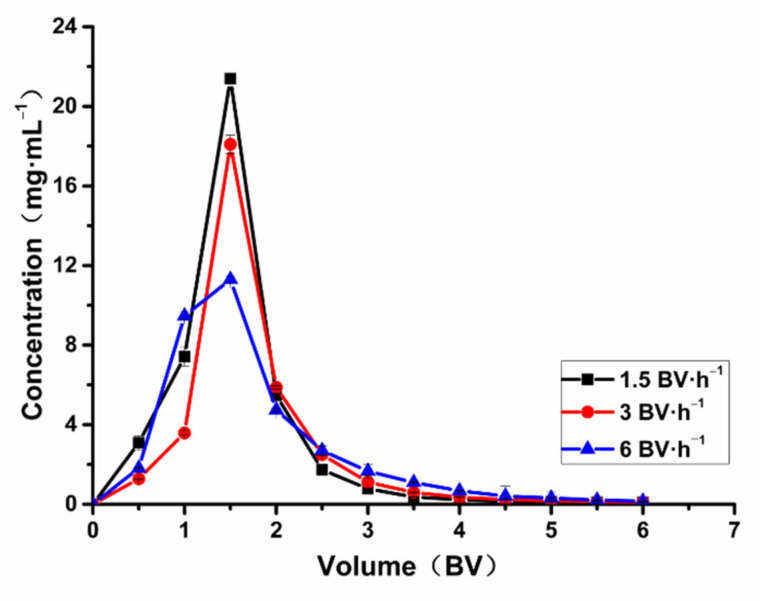
Leakage curve.

To enhance the desorption capacity and reduce consumption of eluent, the flow rates studied in this experiment were 1.5, 3, and 6 BV·h^−^^1^. An ethanol-water solution (60%) was used to elute the total flavonoids. From Figure 8, we can see that the concentration of total flavonoids was highest at 1.5 BV·h^−1^. If the eluting velocity was 6 BV·h^−1^, the effluent concentration was lower than 1.5 and 3 BV·h^−1^, perhaps because the total flavonoids adsorbed on the HPD-600 macroporous resin column could not be eluted in time. Within the range of 12 elution BV, the peaks are narrow and high at 1.5 and 3 BV·h^−^^1^, indicating that low flow rates are more conducive to the elution of total flavonoids, but there is little difference between 1.5 BV·h^−1^ and 3 BV·h^−1^ on the elution capacity. Considering saving time and improving efficiency, 3 BV·h^−1^ was chosen as a suitable desorption flow rate. Thus, 3 BV·h^−1^ was chosen as a suitable desorption flow rate. When the desorption flow rate is 3 BV·h^−1^, the concentration of total flavonoids was unchanged basically at 4 BV elution volume. Thus, the desorption volume was determined as 4 BV.

### 2.8. Validation Experiments

The purification and recovery are two major indexes of verifying condition of purification. The results of the confirmatory test showed that the purity of EAS was 28.79% ± 1.23%, and the purity of ASTFs was 50.57% ± 1.54%. The recovery of total flavonoids was 76.4% ± 2.1%.

### 2.9. Antioxidant Activities Test

To evaluate the antioxidant capacity before and after purification through several indicators, DPPH, ABTS, hydroxyl radicals, and reducing power, the reaction principles of four methods have a difference. The methods of ABTS and hydroxyl radical were based on transfer of electron, and DPPH is due to electron transfer or accepting hydrogen atoms [28]. The detection of iron reduction ability is also based on electron transfer; Fe^2+^ can promote the conversion of hydroxyl radicals and accelerate the oxidation reaction [29]. Antioxidants can reduce Fe^2+^ ions to Fe^3+^, reducing the oxidation reaction. In addition, due to the hydrophobicity of DPPH, it may affect the scavenging ability of hydrophilic compounds, so the anti-oxidant ability can be fully evaluated by combining with water-soluble ABTS [30]. These several measurement methods are complementary, and the measurement method is simple and fast [31], so these several indicators are used to evaluate the antioxidant capacity of total flavonoids before and after purification. L-ascorbic acid was chosen as the positive control because it has a strong antioxidant effect, which has been confirmed in many in vivo and in vitro experiments [32].

**Figure 9:**
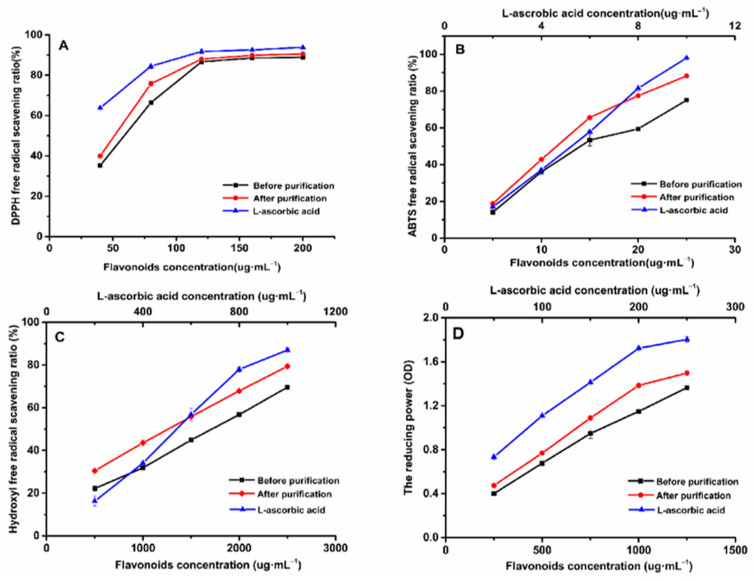
DPPH free radical scavenging ratio (**A**); ABTS free radical scavenging ratio (**B**); hydroxyl free radical scavenging ratio (**C**); and the reducing power (**D**) of L-ascorbic acid, ASTFs, and EAS.

The method of scavenging DPPH free radicals has been widely used to study the antioxidant capacity of a substance [33]. Figure 9A shows that, regarding the DPPH scavenging ratio in the concentration range of 40 ug·mL^−1^ to 120 ug·mL^−1^, the DPPH scavenging ratio was increased with the increasing concentration of EAS and ASTFs, and, when the concentration of EAS and ASTF was 200 ug·mL^−1^, the scavenging ratio reached 88.91% and 90.56%. The IC_50_ of L-ascorbic acid, ASTFs, and EAS was 26.125, 41.694, and 54.828 μg·mL^−1^, respectively. At the same concentration, the scavenging of ASTFs was always stronger than EAS but lower than L-ascorbic acid, which is probably because ASTFs provides more hydrogen atom than EAS, which can be combined with a DPPH radical, resulting in the reduction of a DPPH radical [34].

In Figure 9B, the IC_50_ of L-ascorbic acid, ASTFs, and EAS was 5.049, 12.283, and 15.831 μg·mL^−1^ in the ABTS scavenging experiment. With the range of 5 μg·mL^−1^ to 25 μg·mL^−1^, the ABTS scavenging ratio of EAS and ASTFs was increased, and the scavenging ratio increased from 18.80% to 88.27%. And the ABTS radical scavenging ratio of EAS was lower than ASTFs, but 25 μg·mL^−1^ASTFs is lower than 10 μg·mL^−1^ L-ascorbic acid (98.09%).

Hydroxyl radicals are the most active oxygen species in the body [35]; they can easily pass through the cell membrane and can react with many biological molecules, such as proteins, DNA, lipids, etc., which can lead to cell death and induce various diseases [36]. It is important to survey the hydroxyl radical scavenging capacity of ASTFs. In a hydroxyl radical scavenging capacity comparison assay from Figure 9C, the scavenging ability of 500–2500 μg·mL^−1^ EAS, ASTFs and 200–1000 μg·mL^−1^ L-ascorbic acid was detected. The scavenging ratio of 2500 μg·mL^−1^ EAS and ASTFs were 69.59%, 79.38%, respectively. The IC_50_ of L-ascorbic acid, ASTFs, and EAS was 0.546, 1.267, and 1.716 mg·mL^−1^. This result shows that the’ scavenging capacity of hydroxyl radicals of ASTFs is stronger than EAS, and the purification has a certain effect.

In addition, the reducing power of a compound is a potential indicator to evaluate the antioxidant capacity [37], and Fe^2+^ can promote the conversion of hydroxyl radicals, thereby accelerating the oxidation reaction. Therefore, it is necessary to investigate whether ASTFs can promote the reduction of Fe^2+^. Figure 9D shows that the reducing power of L-ascorbic acid was stronger than that of ASTFs and EAS, while ASTFs had a stronger reducing power than EAS. Compared with EAS, ASTFs are more capable of reducing divalent Fe^2+^ to Fe^3+^, showing stronger oxidation resistance.

Four antioxidant indexes were measured in the antioxidant test; Figure 9 shows that the order of the antioxidant capacity of L-ascorbic acid, ASTFs, and EAS was L-ascorbic acid > ASTFs > EAS. Thus, compared with EAS, the ASTFs purified by HPD-600 had a better antioxidant ability.

### 2.10. UHPLC–MS/MS Analysis of the Enriched Flavonoid and Crude Flavonoid Extract of AS

The composition of ASTFs before and after purification was identified by UHPLC–MS/MS. It can be seen that the content of seven flavonoids increased. As shown in Table 5, the contents of seven kinds of flavonoids—protocatechuic acid, isofraxidin, 6″,6″-dimethylpyrano[2″,3″:7,8]flavone, umbelliferone, luteolin, baicalin, and baicalein—purified using HPD-600 were increased in by 213.75, 13.09, 1.44, 162.29, 161.56, 107.08, and 28.29 times, respectively. The characterization of the increase in these active flavonoid components will be beneficial for the study of AS.

## 3. Materials and Methods

### 3.1. Reagents and Instruments

Macroporous resins (CAD-40, DM301, D-101, HPD-600, S-8, and AB-8) were purchased from Dong Hong Chemical Co., Ltd. (Cangzhou City, China); 95% ethanol, sodium hydroxide, hydrochloric acid, sodium nitrite, and aluminum nitrate were purchased from Sigma-Aldrich Co. (St. Louis, MO, USA). Disodium hydrogen phosphate dodecahydrate, potassium phosphate monobasic, hydrogen peroxide, sulfate heptahydrate, and salicylic acid were purchased from Yantai Yuandong Fine Chemical Co., Ltd. (Laiyang, China). Potassium ferricyanide, potassium persulfate, iron chloride, and trichloroacetic acid were purchased from Aladdin Industrial Corporation Qiang Rd, Nanqiao Town, Fengxian Shanghai, China. The rutin standard was purchased from Shanghai Yuanye Bio-Technology Co., Ltd. (Shanghai, China). Double-distilled water was prepared using a Milli-Q water purification system.

The other chemical reagents were of analytical grade and were purchased from Sinopharm Chemical Reagent Company (Shanghai, China).

Instruments included: UV-5900 Ultraviolet Visible Spectrophotometer (Shanghai Yuanxi Instrument Co., Ltd., Shanghai, China); BT100-2J Peristaltic Pump Driver (Baoding Lange Constant Current Pump Co., Ltd., Baoding, China); PE3000B-type rotary evaporator (Shanghai Yarong Biochemical Instrument Factory, Shanghai, China); SB-600DTY ultrasonic multi-frequency cleaning machine (Ningbo Xinzhi Biological Technology Co., Ltd., Ningbo, China); F-80 Portable Vacuum Pump (Tianjin Fufang Optoelectronic Technology Development Co., Ltd., Tianjin, China); HZQ-F160A constant temperature oscillator (Shanghai Yiheng Scientific Instrument Co., Ltd., Shanghai, China); SHIMADZU Ultra Performance Liquid Chromatograph (LC-30) (Beijing Keyi Hengda Technology Co., Ltd., Beijing, China); SCIEX 5600+ mass spectrometer; and SHIMADZU InerSustain C18 (100 mm × 2.1 mm, 2 µm) (Beijing Keyi Hengda Technology Co., Ltd., Beijing, China).

### 3.2. Experimental Methods

#### 3.2.1. Pretreatment of Total Flavonoids Extract from *Acanthopanax senticosus*

*Acanthopanax senticosus* powder was extracted with 60% ethanol at a solid-liquid ratio of 1:40, and then sonicated (at 60 °C for 65 min) using a SB-600DTY ultrasonicator and filtered. After that, the filtrate was collected, and the crude extracts was concentrated via rotary evaporation under a partial vacuum at 60 °C. After being freeze-dried, the extracts were stored in the fridge at −20 °C.

#### 3.2.2. Determination of Total Flavonoid Content

The total flavonoid content was measured by the method formerly proposed by Zhang [38], with slight modifications. The solution to be measured (1 mL) was placed in a 25 mL Erlenmeyer flask and then mixed with 1 mL of sodium nitrite solution (10%); after 6 min of standing, 1 mL aluminum nitrate (5%) was added, and the mixture was allowed to stand for 6 min before adding 5 mL sodium hydroxide (4%). The mixture was then diluted to 25 mL with ethanol (60%). After 15 min, the absorbance intensity at 510 nm was recorded by UV–vis spectrophotometry using a UV-5900 spectrophotometer. The ethanol solution (60%) was used as the blank and rutin as the standard. The total flavonoid content is presented as the amount of rutin in 1 mL of crude extract (mg·mL^−1^).

#### 3.2.3. Pretreatment of Macroporous Resin

Macroporous resin needs to be pretreated before use, according to a method described by Liang with slight modification [39]. The macroporous resin was soaked in 95% ethanol for 24 h before being washed using distilled water without alcohol and sequentially soaked in 5% NaOH (*m*/*v*) and HCL (*v*/*v*) solutions. Then, it was washed with distilled water until it is neutral. The macroporous resin was then soaked in anhydrous ethanol and washed with water again to remove any traces of alcohol.

#### 3.2.4. Screening of Macroporous Resins

In selecting the optimum macroporous resin, we mainly considered the adsorption capacity, desorption ratio, and desorption capacity [40]. Six kinds of macroporous resins were placed into conical bottles. Next, 25 mL of a solution containing 4.28 mg·mL^−^^1^ ASTFs was added to the HZQ-F160A constant-temperature oscillator for 24 h (30 °C, 100 rpm), and the supernatant was centrifuged. Then, the resin was washed twice with distilled water. The washed resin was shaken for 24 h (30 °C, 100 rpm) in 50 mL of 60% (*v*/*v*) ethanol. The supernatant was centrifugated to determine absorbance after adsorption and desorption, and then the concentration of flavonoids was calculated. All experiments were independently repeated three times, simultaneously. The adsorption capacity, desorption ratio, and desorption capacity of various resins were calculated using the following formulas.

The adsorption capacity:

(1) $$q_{t}=\frac{\left(C0-C1\right)\times V1}{M}.$$

The desorption capacity:

(2) $$A=\frac{C2\times V2}{M}.$$

The desorption ratio:

(3) $$B=\frac{C2\times V2}{\left(C0-C1\right)\times V1}\times 100\%,$$

where *c*_0_ (mg·mL^−1^) indicates the concentration of ASTFs before adsorption, *c*_1_ (mg·mL^−1^) indicates the ASTFs concentration of the sample solution after adsorption, *c*_2_ represents the concentration of flavonoids after desorption, and *V*1 (mL) and *V*2 (mL) represent the bulk of the sample solution and desorption solution, respectively. *M* (g) is the dry weight of the microporous resin.

#### 3.2.5. Adsorption Kinetics of ASTFs on HPD-600 Resins

A total of 2.00 g of HPD-600 resin was placed in a 100 mL conical bottle, and then 4.153 mg·mL^−1^ of *Acanthopanax senticosus* total flavonoids with 30 mL was added to oscillate and adsorbed on a constant-temperature oscillator (30 °C/100 rpm). Next, a 1 mL aliquot of the total flavonoid solution was removed every 30 min to measure the contents and calculate the adsorption ratio. The adsorption kinetics curve of HPD-600 was drawn using the data obtained. The adsorption kinetic data were fitted by means of quasi-first-order kinetics model, quasi-second-order kinetics model, and intraparticle diffusion model, respectively [22,41,42].

The quasi-first-order kinetic equation:

(4) $$\ln\left(q_{e}-q_{t}\right)=\ln q_{e}-k_{1}t.$$

The quasi-second-order kinetic:

(5) $$\frac{t}{q_{t}}=\frac{1}{k_{2}q_{e}{}^{2}}+\frac{t}{q_{e}}.$$

The in-particle diffusion model:

(6) $$q_{t}=k_{p}t^{0.5},$$

where *k*_1_ and *k*_2_ (g·mg^−1^·min^−1^) are equation constants and represent the rate of the quasi-first-order kinetic model and quasi-second-order kinetic model, respectively. *k_p_* (mg·g^−1^·min^−0.5^) and *I* are diffusion equation constants.

#### 3.2.6. Adsorption Isotherms of ASTFs on HPD-600 Resins

A total of 1 g of HPD-600 macroporous resin was precisely weighed and placed into 100 mL conical bottles. The crude extract of ASTFs 25 mL was added to an oscillator at 25, 30, and 35 °C. The total flavonoid content of the supernatant was determined after shaking for 24 h at different temperatures, and the adsorption isotherms were obtained. For the purpose of comprehending the adsorption mechanism of the ASTFs on HPD-600 resin, the adsorption isotherm model was selected for line fitting. The adsorption equation of adsorption isotherm can quantitatively explain the adsorption of flavonoids with different initial concentrations in HPD-600 resin. The adsorption isotherms were fitted using the most common Freundlich adsorption model, Langmuir adsorption model, and Temkin adsorption model to describe their adsorption mechanisms. The three adsorption isotherm equations are as follows:

Freundlich adsorption model:

(7) $$\ln q_{e}=\frac{1}{n}\ln C_{e}+\ln K_{F}.$$

Langmuir adsorption model:

(8) $$\frac{C_{e}}{q_{e}}=\frac{1}{K_{L}q_{m}}+\frac{C_{e}}{q_{m}}.$$

Temkin adsorption model:

(9) $$q_{e}=B_{T}\ln K_{T}+B_{T}\ln C_{e},$$

where *n* and *K_F_* [(mg·g^−1^) (mL·mg^−1^)^1/*n*^] are the Freundlich equation constants and *K_F_* reflects the adsorption capacity, *q_m_* (mg·g^−1^) represents the saturated adsorption capacity, *K_L_* (mL·mg^−1^) is the Langmuir equation constant, and *K_T_* (mL·mg^−1^) and *B_T_* (J·mol^−1^) are the Temkin equation constants.

#### 3.2.7. Adsorption Thermodynamics of ASTFs on HPD-600 Resins

The analysis of the thermodynamic parameters of adsorption enabled us to understand the thermodynamic behavior of resin and the internal energy change after adsorption [43,44]. There are three main parameters: enthalpy change ΔH (KJ·mol^−1^), free energy change ΔG (KJ·mol^−1^·K^−1^), and entropy change ΔS (KJ·mol^−1^).

The enthalpy change ΔH was calculated according to Clausius–Clapeyron equation:

(10) $$\ln C_{e}=-\ln k_{0}+\frac{\Delta H}{\mathrm{RT}}.$$

The free energy ΔG was calculated according to Gibbs’ equation and adsorption isotherm constant:

(11) $$\Delta G=-\mathrm{RT}\int_{0}^{X}q\frac{d_{x}}{x}.$$

The Freundlich Equation (8) can be substituted into Equation (11) if the fitting result of the adsorption isotherm is in accordance with the Freundlich model, and the following can be obtained:

(12) ΔG=−nRT.

The entropy change ΔS is calculated according to the Gibbs–Helmholtz equation:

(13) $$\Delta S=\frac{\Delta H\;-\;\Delta G}{\Delta T}.$$

*C_e_* is the counterpoise mass concentration of the total flavonoid solution of *Acanthopanax senticosus* under the same amount of adsorption in mg·g^−1^; R is the gas constant of 8.314 J·moL^−1^·K^−1^; T represents the thermodynamic temperature; and *n* is the characteristic parameter in the Freundlich adsorption isotherm model.

#### 3.2.8. Static Adsorption and Desorption Experiments

##### Sample Liquid pH Investigation

We use 1% HCl (*v*/*v*) and 1% NaOH (*m*/*v*) to adjust the pH of a concentrated extract of AS to 1, 2, 3, 4, 5, 6, and 7, respectively. Resin HPD-600 (2 g) was added into the solution and shaken at 30 °C and 100 rpm for 3 h, and the adsorption capacity and ratio were calculated based on the content of flavonoids.

##### Ethanol Concentration on Desorption Capacity

Ethanol and water were mixed at several different ratios (30%, 40%, 50%, 60%, 70%, 80%, 90%) and used as eluents to determinate the desorption capacity after complete absorption of the flavonoids onto the HPD-600 resins, and the desorption efficiency for different ethanol/water ratios were then investigated.

#### 3.2.9. Dynamic Adsorption and Desorption Experiments

##### Effect of Sample Condition

A glass column with a 16 mm inner diameter and a 40 cm length filled with HPD-600 resin was used to execute a dynamic adsorption and desorption process; the bed volume was 20 mL in this test. ASTFs (9 mg·mL^−1^) was loaded at different flow rates (1.5, 3, and 6 BV·h^−1^). The optimal flow rate was chosen according to the adsorption capacity of the resin column and the determined dynamic leakage curves. The volume of the effluent was used as the horizontal coordinate, while the mass concentration of ASTFs effluent was used as the vertical coordinate.

##### Effect of Elute Condition

The desorption process was operated by washing the HPD-600 resin column with distilled water twice, followed by desorption with 60% ethanol at different flow rates (1.5, 3, and 6 BV·h^−1^).

We collected a tube of outflowing fluid every 10 mL for the determination of the flavonoid content. When drawing the dynamic elution curve, the effluent volume was the horizontal coordinate, and the concentration of total flavonoids in the solution was the vertical coordinate.

### 3.3. Validation Test

The total flavonoids of *Acanthopanax senticosus* were enriched according to the above optimized conditions, the recovery rate of flavonoids was calculated, and the purification of EAS and ASTFs was compared. After freeze-drying the EAS and ASTFs solutions, weigh the dry powder of a certain mass), then dissolve it to the same volume with ethanol, measure the OD, calculate the flavonoid mass in EAS and ASTFs, and take the mass of flavonoids in the dry powder mass. The percentage is purity.

(14) $$P1=\frac{m1}{m2}\times 100\%,$$

(15) $$P2=\frac{m3}{m4}\times 100\%,$$

(16) $$R=\frac{C1\times V1}{C2\times V2}\times 100\%,$$

where *P*1 expresses the purity of EAS, *P*2 expresses the purity of ASTFs, and *R* expresses the recovery ratio. *m*1 = the quality of flavonoids in EAS; *m*2 = the quality of EAS; *m*3 = the quality of flavonoids in ASTFs; *m*4 = the quality of ASTFs. *C*1 = the concentration of flavonoid in EAS; *V*1 = the loaded sample volume; *C*2 = the concentration of flavonoid in ASTFs; *V*2 = the eluted volume.

### 3.4. Antioxidant Activities Test

To verify the purification capacity of HPD-600 resin, the scavenging rates of DPPH, ABTS, and hydroxyl radical and the reducing power were used as indicators to compare the antioxidant ability of EAS and ASTFs. The antioxidant capacity was estimated from the IC50, which is the concentration of a sample when the inhibition ratio is 50%. The DPPH scavenging assay of EAS and ASTFs were tested according to the procedure described by Andrade [45], where 1 mL of samples of different concentrations (40, 80, 120, 160, 200 μg·mL^−1^) or L-ascorbic acid solution were taken, and 2 mL of DPPH solution (0.2 mmol·L^−1^) was added, mixed well, and allowed to react at room temperature in the dark for 30 min. We then measured the absorbance at 517 nm [34]. The DPPH radical scavenging activity was calculated using Equation (17):

(17) $$\mathrm{DPPH}\;\mathrm{radical}\;\mathrm{scavenging}\;\mathrm{ratio}\;(\%)=\frac{A1-\left(A2-A3\right)}{A1}100\%,$$

where *A*1 is the absorbance of the absolute ethyl alcohol with DPPH, *A*2 is the absorbance of the sample with DPPH, and *A*3 is the absorbance of the sample without DPPH.

The ABTS free radical scavenging assay was performed according to the method described by Ullah [46], with some modifications. First, 7 mM ABTS and 2.5 mM potassium persulfate solution were mixed in equal volumes, and then the reaction was kept at room temperature, avoiding light, for 14 h. Additionally, the mixed solution was diluted to an absorbance of 0.7 ± 0.02 at 734 nm. Next, 0.2 mL sample solution or L-ascorbic acid solution of different concentrations are mixed with 0.8 mL ABTS evenly, and then measuring the absorbance at 734 nm after reaction of 6 min.

(18) $$\mathrm{ABTS}\;\mathrm{radical}\;\mathrm{scavenging}\;\mathrm{ratio}\;(\%)=\frac{A1-\left(A2-A3\right)}{A1}100\%,$$

where *A*1 is the absorbance of the absolute ethyl alcohol with ABTS, *A*2 is the absorbance of the sample with ABTS, and *A*3 is the absorbance of the sample without ABTS.

The hydroxyl radical scavenging assay was carried out according to the following method: 1 mL sample or L-ascorbic acid solution of different concentrations was added in a centrifuge tube; then, add 1 mL of 3 mM FeSO_4_ solution, 1 mL of 1 mM H_2_O_2_ solution, and add 1 mL of 3 mM salicylic acid alcohol solution, in turn. The absorbance of these mixtures was measured at 510 nm after left in 37 °C water for 30 min.

(19) $$\mathrm{Hydroxyl}\;\mathrm{radical}\;\mathrm{scavenging}\;\mathrm{ratio}\;(\%)=\frac{A1-\left(A2-A3\right)}{A1}100\%,$$

where *A*1 is the absorbance of the blank control, *A*2 is the absorbance of the sample, and *A*3 is the absorbance of the sample without H_2_O_2_.

We detected the reducing power according to the test method described by Chen [47] and determined the reducing power according to the OD value. All the above tests used ethanol as a blank control and L-ascorbic acid as a positive control.

### 3.5. UHPLC–MS/MS Analysis of Crude and Total Flavonoid-Enriched Extracts

This experiment used a SHIMADZU ultra-high performance liquid chromatograph (LC-30) (Tokyo, Japan) linked with a SCIEX5600 mass spectrometer (Tokyo, Japan) (Hybrid Quadrupole-TOF LC/MS/MS Mass Spectrometer), respectively, using electrospray ion source positive and negative ion mode. The SHIMADZU InerSustain C18 chromatographic column (100 mm × 2.1 mm, 2 µm) (Tokyo, Japan) was used to separate and detect the flavonoids in the purified and crude extracts of *Acanthopanax senticosus*. The working temperature was 30 °C, and the flow rate was 1 mL·min^−1^. Solvent A was acetonitrile, and solvent B was 0.1% HCOOH-H_2_O. The elution was completed using the following gradient: 0–2 min 95% B, 2–4 min 95–80% B, 4–12 min 80–75% B, 12–14 min 75–54% B, 14–26 min 54–0% B, 26–28 min 0% B, 28–29 min 0–95% B, 29–30 min 95% B.

### 3.6. Statistical Analysis

The test data were collected in Microsoft Excel 2013. All the experiments were independently executed three times. All the presented values are means ± SD. Tukey’s HSD test (*p* < 0.01 or *p* < 0.001) was carried out using the SPSS statistics program. The figures were built using Origin 2019b (Origin Lab Corporation, Northampton, MA, USA).

## 4. Conclusions

A method for purifying ASTFs from EAS using macroporous resin was established. The results of the adsorption/desorption features and kinetic conclusion all illustrated that HPD-600 was the best choice for purifying ASTFs from EAS among the six tested resins. Using this resin, the purity of the purified total flavonoids was 50.57% ± 1.54%, which was 1.76-fold higher than the concentration in EAS, and the recovery rate of the total flavonoids reached 76.4% ± 2.1%. The antioxidant activity test showed that the antioxidant capacity of ASTFs was higher than that of EAS, but it could not reach that of L-ascorbic acid. We found that the contents of seven flavonoids in the purification were higher than in the crude extract. The Freundlich equation and pseudo-second-order model could be used to accurately formulate static adsorption and dynamic adsorption, respectively. The adsorption data were analyzed, illustrating that the adsorption kinetics data had a strong correlation with the quasi-second-order kinetic equation and Freundlich isothermal equation, as well as that the adsorption process was spontaneous and endothermic. This further theoretically explains and supports the kinetic mechanism underlying the HPD-600 purification of ASTFs. For the next step, we need to scale up the HPD-600 purification of total flavonoids from *Acanthopanax senticosus* to determine whether the current laboratory purification method can be applied to clinical practice and try to provide improved purification technology and correct theoretical support for the practical application of ASTFs.

## Figures and Tables

**Table 1:** Physical properties of the six types of macroporous resins assessed in this study.

Polarity	Resin Type	Surface Area/m^2^·g^−1^	Average Pore/A^°^
D101	No-polar	500~550	90~100
AB-8	Weak polar	480~520	130~140
CAD-40	Middle polar	450~500	70~80
DM301	Middle polar	330~380	130~170
HPD-600	Polar	550~600	80
S-8	Polar	100~120	280~300

**Table 2:** Kinetic model fitting equations and model parameters.

Dynamics Model	Kinetic Equation	Parameters
The pseudo-first-order model	ln(*q_e_* − *q_t_*) = −0.0145*t* + 2.488	*k*_1_ = 0.0145
		*R*^2^ = 0.9616
		*q_e_* = 12.037 mg·g^−1^
The pseudo-second-order model	*t*/*q_t_* = 0.0341*t* + 0.3004	*k*_2_ = 0.0031 g·(mg·min)^−1^
		*R*^2^ = 0.9995
		*q_e_* = 29.326 mg·g^−1^
Intra-particle diffusion model	*q_t_* = 1.72*t*^0.5^ + 10.22	*k_p_* = 1.0062 mg·(g·min)^−1/2^
		*R*^2^ = 0.9739
		*I* = 10.22
	*q_t_* = 0.47*t*^0.5^ + 21.96	*k_p_* = 1.0062 mg·(g·min)^−1/2^
		*R*^2^ = 0.8541
		*I* = 21.96
	*q_t_* = 0.07*t*^0.5^ + 27.28	*k_p_* = 1.0062 mg·(g·min)^−1/2^
		*R*^2^ = 0.8958
		*I* = 27.28

**Table 3:** Adsorption isotherm models fitting regression equations and adsorption parameters at different temperatures.

Model	T/(°C)	Equations	Parameters		
			*K_F_* [(mg·g^−1^) (mL·mg^−1^)^1/*n*^]	*n*	*R* ^2^
Freundlich	25	ln*q_e_* = 0.8435ln*C_e_* + 2.295	9.926	1.1855	0.9772
	30	ln*q_e_* = 0.7898ln*C_e_* + 2.465	11.761	1.2661	0.9788
	35	ln*q_e_* = 0.8708ln*C_e_* + 2.8538	17.354	1.1484	0.9908
			*K_L_* (mL·mg^−1^)	*Q_m_* (mg·g^−1^)	*R* ^2^
Langmuir	25	*C_e_*/*q_e_* = 0.0056*C_e_* + 0.0969	0.0578	178.57	0.8017
	30	*C_e_*/*q_e_* = 0.0063*C_e_* + 0.0827	0.0762	158.73	0.9319
	35	*C_e_*/*q_e_* = 0.0024*C_e_* + 0.0571	0.0434	416.67	0.8316
			*K_T_* (L·mg^−1^)	*B_T_* (J·mol^−1^)	*R* ^2^
Temkin	25	*q_e_* = 25.788ln*C_e_* + 2.7832	1.1139	25.788	0.9667
	30	*q_e_* = 26.741ln*C_e_* + 4.5813	1.1869	26.741	0.9694
	35	*q_e_* = 50.038ln*C_e_* + 1.2145	1.0246	50.038	0.9456

**Table 4:** HPD-600 thermodynamic parameters of total flavonoids from AS.

Adsorption Capacity *q_e_*/(mg·mL^−1^)	Enthalpy Change ΔH/(kJ·mol^−1^)	Free Energy Changes ΔG/(kJ·mol^−1^)			Entropy Change ΔS/J·(mol·K)^−1^		
		25 °C	30 °C	35 °C	25 °C	30 °C	35 °C
20	58.2429				205.5695	202.5583	198.5412
30	59.3270				209.2122	206.136	202.0647
40	60.0961	−2.9385	−3.1384	−2.8467	211.7962	208.6738	204.5641
50	60.6922				213.7992	210.641	206.5014
60	61.1802				215.4389	212.2515	208.0875

**Table 5:** Comparison of total flavonoids of *Acanthopanax senticosus* before and after purification.

Average Rt (min)	Adduct Type	Contents	Formula	SMILES	Class	Content (%)	
						Before Purification	After Purification
5.255	[M − H]^−^	Protocatechuic acid	C_7_H_6_O_4_	OC(=O)C1=CC(O)=C(O)C=C1	Flavones	0.0538	11.4892
6.346	[M + H]^+^	Isofraxidin	C_11_H_10_O_5_	O=C1OC=2C(OC)=C(O)C(OC)=CC2C=C1	7-hydroxycoumarins	0.1130	1.4791
7.839	[M + H]^+^	6″,6″-Dimethylpyrano[2″,3″:7,8]flavone	C_20_H_16_O_3_	O=C1C=C(OC=2C1=CC=C3OC(C=CC32)(C)C)C=4C=CC=CC4	Pyranoflavonoids	0.1686	0.2432
8.001	[M − H]^−^	Umbelliferone	C_9_H_6_O_3_	O=C1OC=2C=C(O)C=CC2C=C1	Flavones	0.0082	1.3348
9.454	[M + H]^+^	Luteolin	C_15_H_10_O_6_	OC1=CC(O)=C2C(=O)C=C(OC2=C1)C1=CC(O)=C(O)C=C1	Flavones	0.0064	1.0353
10.163	[M + H]^+^	Baicalin	C_21_H_18_O_11_	O=C(O)C4OC(OC1=CC=2OC(=CC(=O)C=2(C(O)=C1(O)))C3=C	Flavone O-glycosides	0.0532	5.6938
15.43	[M − H]^−^	Baicalein	C_15_H_10_O_5_	O=C1C=C(OC2=CC(O)=C(O)C(O)=C12)C=3C=CC=CC3	Flavones	0.0870	2.4605

## Data Availability

Not applicable.
